# Species-Specific Metabolite Profiles and Biological Activities of Bulgarian *Thymus* Species from Section *Hyphodromi*

**DOI:** 10.3390/plants15060927

**Published:** 2026-03-17

**Authors:** Denitsa Kancheva, Milena Nikolova, Vasil Georgiev, Borislav Georgiev, Elina Yankova-Tsvetkova, Ina Aneva

**Affiliations:** 1 Institute of Biodiversity and Ecosystem Research, Bulgarian Academy of Sciences, 1113 Sofia, Bulgaria; mtihomirova@gmail.com (M.N.); bobogeorgiev5@gmail.com (B.G.); e_jankova@abv.bg (E.Y.-T.); 2 Laboratory of Cell Biosystems, Institute of Microbiology, Bulgarian Academy of Sciences, 139 Ruski Blvd., 4000 Plovdiv, Bulgaria; vasgeorgiev@gmail.com; 3 Bulgarian Academy of Sciences, “15 Noemvri” Str., 1040 Sofia, Bulgaria

**Keywords:** Bulgarian endemics, phenolic compounds, terpenes, antioxidant activity, acetylcholinesterase inhibition

## Abstract

This study investigates the phytochemical composition and biological activity of eight *Thymus* species distributed in Bulgaria, with a focus on taxa from section *Hyphodromi*. High-performance liquid chromatography (HPLC) and gas chromatography–mass spectrometry (GC–MS) were used to characterize the methanolic (MeOH) and diethyl ether (Et_2_O) extracts, respectively. MeOH extracts revealed high concentrations of phenolic acids, particularly rosmarinic acid, salicylic acid, and flavonoid glycosides, with *T. atticus*, *T. jalasianus*, and *T. leucotrichus* showing the most diverse profiles. Et_2_O extracts were dominated by triterpenic acids (ursolic and oleanolic) and monoterpenes such as thymol and carvacrol, especially in *T. zygioides* and *T. leucotrichus*. All MeOH extracts exhibited significant antioxidant activity in the DPPH assay (IC_50_ < 50 µg/mL), with *T. jalasianus* and *T. atticus* demonstrating the strongest effects. Radical scavenging potential generally followed the trend of total phenolic content. Moderate acetylcholinesterase-inhibitory activity was observed only in *T. zygioides* and *T. leucotrichus*. The study reports for the first time data on the biological activity and metabolic composition of extracts from endemic and rare species and from the species of Bulgarian origin. The results provide new data on the phytochemical composition and in vitro antioxidant and acetylcholinesterase-inhibitory activities of selected *Thymus* species, contributing to the characterization of their overall in vitro biochemical profiles.

## 1. Introduction

The genus *Thymus* L. (Lamiaceae) encompasses over 200 species and is one of the most taxonomically and phytochemically complex genera among medicinal and aromatic plants. Distributed mainly in the Mediterranean region and southwestern and Central Asia, its members are well known for their adaptive plasticity and extensive chemodiversity [1]. Bulgaria is recognized as a center of diversification for *Thymus*, hosting more than 20 native taxa, including a number of local and Balkan endemics [2]. Section *Hyphodromi* forms a prominent group within the Bulgarian flora and includes species with restricted distribution and ecological specialization [3,4].

Species of the genus *Thymus* are widely used in traditional and modern herbal medicine due to their essential oils and polar bioactive constituents. Phytochemically, they are rich in monoterpenes, phenolic acids, flavonoids, and triterpenes, which contribute to various biological effects, including antimicrobial, antioxidant, and anti-inflammatory activities. Acetylcholinesterase-inhibitory activity has also been reported for certain *Thymus* species and individual constituents in previous in vitro studies [5,6,7,8]. The essential oils of *Thymus* species are dominated by chemotypes containing thymol, carvacrol, linalool, or geraniol, which are often used as markers in ecological and pharmacognostic studies [9,10,11]. Alongside these volatile compounds, polar metabolites such as rosmarinic, caffeic, and ferulic acids, as well as flavonoids like luteolin, apigenin, and rutin, play a crucial role in the plant’s antioxidant mechanisms [11,12,13]. Triterpenic acids—especially ursolic and oleanolic acid—have also been highlighted for their anti-inflammatory and antimicrobial activities, while acetylcholinesterase-inhibitory activity has been reported mainly in the context of isolated compounds and in vitro assays [14,15,16,17].

Although our previous research [18,19,20] has indicated the presence of potentially valuable bioactive compounds in lesser-known or narrowly distributed species such as *T. atticus Čelak.*, *T. stojanovii* Degen, *T. perinicus (Velen.)* Jalas, and *T. jalasianus* Stoyanov & Marinov, a comprehensive phytochemical profiling with biological activity assessment of Bulgarian species within section *Hyphodromi* has not yet been reported. Given the rising interest in plant-based bioactive substances, the systematic evaluation of endemic *Thymus* species from this section is of both scientific and practical importance. In this context, the present study aims to (i) analyze the chemical composition of methanolic (MeOH) and diethyl ether (Et_2_O) extracts of selected Bulgarian *Thymus* species from section *Hyphodromi* and (ii) evaluate their antioxidant and acetylcholinesterase (AChE)-inhibitory activities through in vitro assays. In the present study, acetylcholinesterase inhibition was included as an exploratory in vitro biochemical screening assay, without implying neuroprotective, ethnopharmacological, or therapeutic relevance. This integrated approach aims to characterize the phytochemical profiles and in vitro biological activities of selected Bulgarian *Thymus* species and to provide a basis for future studies using biologically relevant experimental models.

## 2. Results

### 2.1. Phytochemical Composition

The phytochemical analysis of both MeOH and Et_2_O extracts from the studied *Thymus* species revealed diverse profiles of primary and secondary metabolites. The results are presented in Table 1 and Table 2.

**Figure 1:**
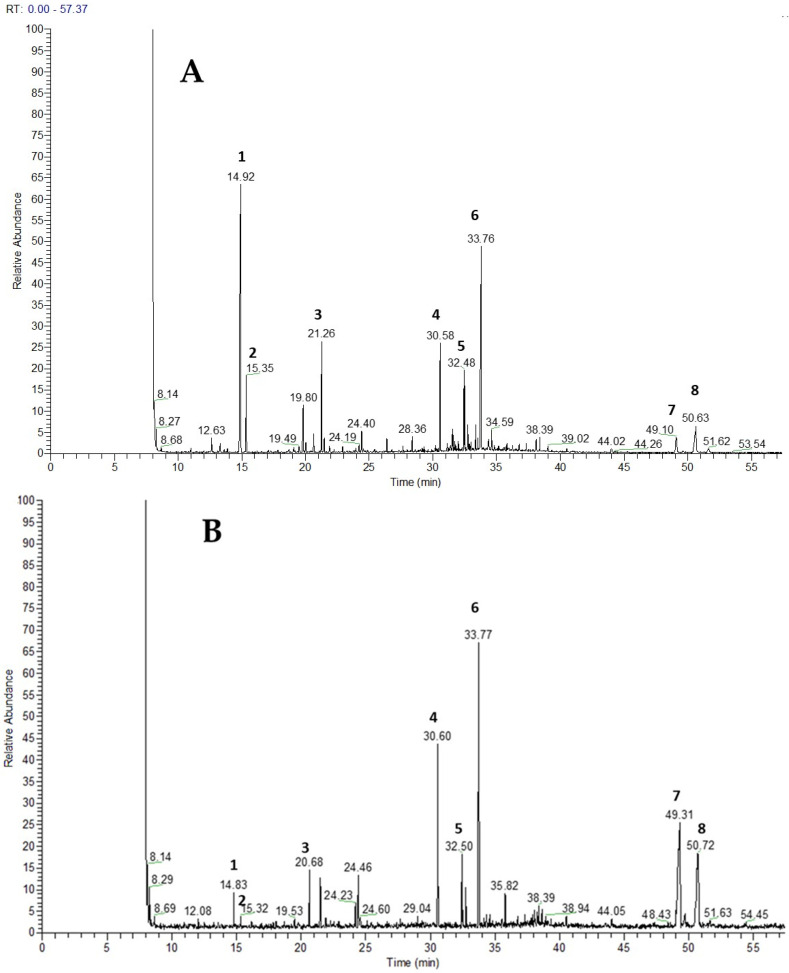
GC/MS chromatograms of Et_2_O extracts: (**A**) *T. leucotrichus* extract; (**B**) *T. striatus* extract; **1**. thymol; **2**. carvacrol; **3**. *tert*-butylhydroquinone; **4**. hexadecanoic acid; **5**. fatty acids; **6**. internal standard nonadecanoic acid; **7**. oleanolic acid; **8**. ursolic acid.

GC–MS analysis revealed triterpenic acids as the dominant constituents of Et_2_O extracts, primarily ursolic and oleanolic acid. The highest amounts of triterpenes were observed in *T. zygioides* and *T. jalasianus*. The highest ursolic acid content was recorded in *T. jalasianus*, while oleanolic acid was most abundant in *T. striatus*. The ursolic/oleanolic acid ratio favored ursolic acid in most species, except for *T. striatus* and *T. atticus* where oleanolic acid prevailed. In addition to triterpenes, the monoterpenes thymol and carvacrol were also consistently detected as major bioactive constituents in all Et_2_O extracts. The highest levels of these compounds were found in *T. leucotrichus* and *T. zygioides*. Representative GC–MS chromatograms of selected species with identified compounds marked are presented in Figure 1. The complete set of chromatograms is provided in the Supplementary Materials.

HPLC analysis of the MeOH extracts identified rosmarinic acid as a major phenolic constituent across most taxa, with particularly high levels in *T. atticus*, *T. aznavourii*, and *T. jalasianus*. Other detected phenolic compounds included *p*-coumaric acid, ferulic acid, salicylic acid, rutin, hesperidin, and quercetin, varying in concentration among species. Of all studied MeOH extracts, the one from *T. atticus* exhibited the most abundant and most diverse phenolic profile, including high levels of rosmarinic acids, *p*-coumaric acid, ferulic acid, rutin, hesperidin, and salicylic acid. All HPLC chromatograms are presented in Supplementary Material.

### 2.2. Phenolic Content and Bioactivity of Methanolic Extracts

The DPPH radical scavenging activity and acetylcholinesterase-inhibitory activity of *Thymus* MeOH extracts and their total phenolic content were evaluated. The results are presented in Table 3 and Table 4.

The total phenolic content and antioxidant activity of the MeOH extracts varied substantially among the studied *Thymus* species. The highest levels of phenolic compounds were found in *T. leucotrichus*, *T. jalasianus*, and *T. aznavourii*, followed closely by *T. atticus*, *T. striatus*, and *T. comptus*. In contrast, *T. perinicus* and *T. zygioides* contained significantly lower amounts of total phenolics, accompanied by higher IC_50_ values in the DPPH assay.

Antiradical activity, assessed through the DPPH assay, showed a positive compliance with total phenolic contents. The most pronounced radical scavenging effects were observed in *T. atticus, T. jalasianus, T. comptus* and *T. aznavourii*, showing lower IC_50_ values in the DPPH assay.

**Figure 2:**
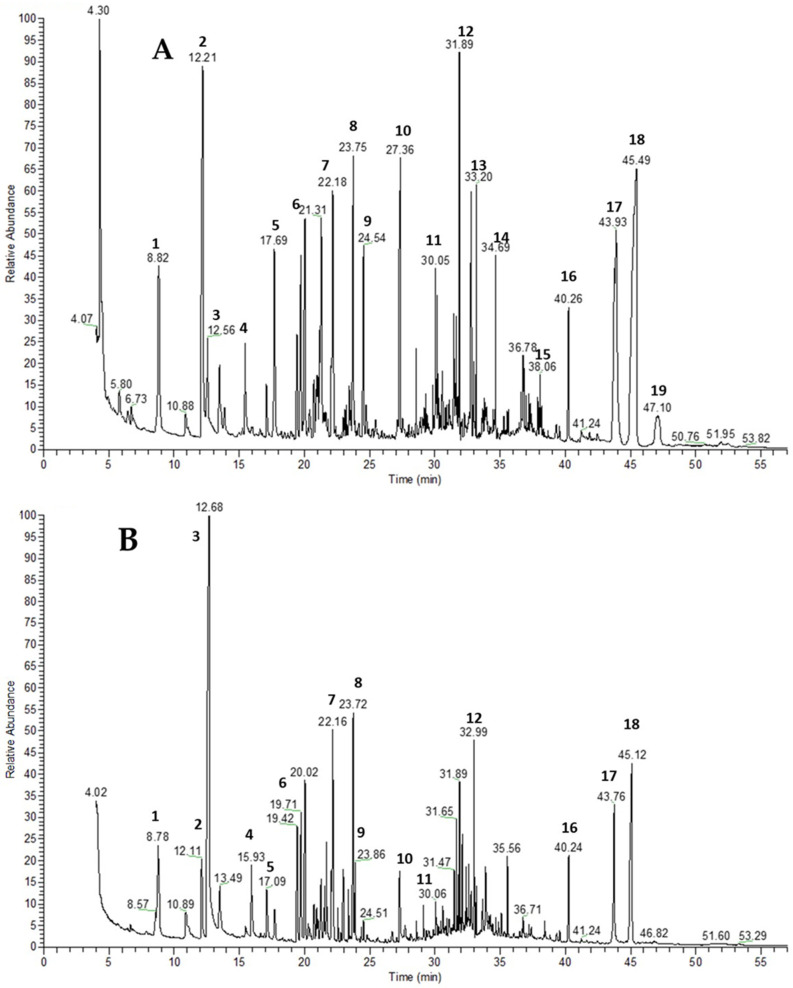
GC/MS chromatograms of MeOH extracts: (**A**) *T. leucotrichus* extract; (**B**) *T. zygioides* extract; **1**. glycerol; **2.** thymol; **3**. carvacrol; **4**. malic acid; **5**. arabitol; **6**. fructose isomers; **7**. glucose; **8**. monosaccharide; **9**. myo-inositol; **10**. hexadecanoic acid; **11**. fatty acids; **12**. sucrose and other disaccharides; **13**. monopalmitin; **14**. glycerol monostearate; **15**. 4-O-Feruloylquinic acid; **16**. rosmarinic acid; **17**. oleanolic acid; **18**. ursolic acid; **19**. micromeric acid.

The acetylcholinesterase-inhibitory activity of the MeOH extracts was generally weak across most species. *T. zygioides* and *T. leucotrichus* exhibited enzyme inhibition to some extent, with IC_50_ values being determinable only when concentrations were increased following initial testing. All other extracts showed no inhibition at the chosen concentrations. To further characterize the chemical composition, the metabolite composition of the MeOH extracts of the two most active species was analyzed additionally using GC–MS, and the results are presented in Table 5. GC/MS chromatograms are presented in Figure 2. High relative abundances of compounds such as oleanolic acid, ursolic acid, thymol, carvacrol, caffeic acid, and rosmarinic acid were found.

## 3. Discussion

### 3.1. Phytochemical Patterns Among Bulgarian Thymus Species

The phytochemical profiling of the Et_2_O extracts revealed that triterpenic acids, monoterpenes, and flavonoid aglycones were the predominant classes of bioactive compounds across the studied *Thymus* species. These findings are in agreement with previous reports indicating that triterpenic acids are major constituents in the exudates of various *Thymus* taxa [19]. Ursolic and oleanolic acids were identified as major triterpene constituents, which is consistent with previous reports for Lamiaceae species. Although these compounds are widespread in medicinal plants and not taxonomically specific, their quantification contributes to the comparative phytochemical characterization of the studied *Thymus* taxa. In most Lamiaceae species, ursolic acid typically predominates over oleanolic acid [21]. This general trend was observed in our study, as well, with the notable exception of *T. striatus* and *T. atticus*, where oleanolic acid was more abundant. The stable accumulation pattern of oleanolic acid in these two taxa, confirmed by earlier studies [19], suggests a chemotaxonomic feature that may have diagnostic relevance.

Monoterpenes such as thymol and carvacrol were also present in significant quantities in the Et_2_O extracts, particularly in *T. zygioides* and *T. leucotrichus*. These compounds are well known for a broad spectrum of biological activities, including antioxidant and antimicrobial effects, as reported in previous in vitro and in vivo studies [22,23,24,25,26]. Their presence in certain *Thymus* species is consistent with the in vitro biochemical profiles observed in the biological assays.

Additionally, flavonoid aglycones identified in the Et_2_O fraction further support the chemical complexity of these *Thymus* species. These compounds, along with triterpenes and monoterpenes, may contribute synergistically to the observed in vitro bioactivities and underline the biochemical relevance of these taxa [27,28,29,30,31,32]. It should be noted that the HPLC analysis applied in this study was based on a targeted approach using selected reference standards and UV detection; therefore, additional phenolic constituents present in the methanolic extracts may remain undetected. Advanced techniques such as HPLC–MS/MS would allow a more comprehensive, untargeted profiling of phenolic constituents and are planned for future investigations. The present study focused on targeted identification of selected, literature-relevant compounds; therefore, additional or structurally novel constituents present in the extracts may remain undetected. Comprehensive untargeted metabolomic approaches, such as HPLC–MS/MS, would be required to establish a complete chemical profile and to identify potentially novel compounds, and are envisaged for future investigations.

### 3.2. Interpretation of Antioxidant and AChE-Inhibitory Activities

The antiradical activity of the studied extracts was similar to that reported for other *Thymus* species [7]. A strong correlation was observed between total phenolic content and DPPH radical scavenging activity. Major components detected in HPLC analyses like rosmarinic acid, *p*-coumaric acid, ferulic acid, hesperidin, rutin, and salicylic acid are associated with the observed antioxidant potential of *T. atticus* extract, in line with previous reports. Notably, *T. jalasianus* and *T. atticus* extracts exhibited a strong antioxidant effect, as well, aligning with their high levels of rosmarinic acid and other phenolic constituents. Our results reinforce the established role of rosmarinic acid and other phenolic acids as well as flavonoids in neutralizing free radicals and are consistent with their reported role in antioxidant activity in vitro [7,12,13,32,33,34,35]. It should be emphasized that the antioxidant activity reported here was evaluated exclusively using a chemical radical scavenging assay and therefore should not be interpreted as evidence of biological antioxidant efficacy in cellular or in vivo systems. Cell-based models represent a more biologically relevant approach for assessing antioxidant responses under physiological conditions, as they account for factors such as bioavailability, metabolism, and cellular signaling pathways [36]. Such experiments were beyond the scope of the present in vitro screening study but represent an important direction for future research. More complex cellular or animal models are indeed more appropriate for evaluating antioxidant efficacy under biological conditions and are beyond the scope of the present in vitro screening study.

Acetylcholinesterase (AChE)-inhibitory activity was generally weak across most species, with the exception of *T. zygioides* and *T. leucotrichus*, which exhibited moderate inhibitory effects when test concentrations were elevated. As seen in Table 2, the largest quantities of thymol and carvacrol were observed in the Et_2_O extracts of *T. zygioides* and *T. leucotrichus*. The additional GC–MS analysis of the MeOH extracts of these species revealed high levels of thymol and carvacrol (Table 5). The fact that these compounds have been previously associated with AChE inhibition [22] is consistent with the acetylcholinesterase-inhibitory activity observed for *T. zygioides* and *T. leucotrichus* under in vitro conditions. Some previous research has indicated that carvacrol is a 10 times more potent AChE inhibitor than thymol [22], and this is consistent with previously reported differences in acetylcholinesterase-inhibitory activity. Besides alkaloids, mono- and triterpenes and their derivatives are among the main classes of natural compounds reported to possess anticholinesterase properties, although generally with lower potency than alkaloid-based or synthetic inhibitors. Examples include thymol, carvacrol, citral isomers, ursolic acid and oleanolic acid, which have been previously discussed in the context of enzyme inhibition. Such compounds were detected in the MeOH extracts of *T. zygioides* and *T. leucotrichus* (Table 5); however, considering their relative abundances in the samples, their concentrations are likely insufficient to produce strong enzyme inhibition, which may explain the moderate activity observed.

It should be emphasized that the observed AchE-inhibitory activity was detected exclusively in vitro and at relatively high extract concentrations; therefore, these results should be regarded as preliminary and not directly indicative of in vivo or cognitive effects.

Oxidative stress occurs when increased reactive oxygen species (ROS) production is present and is associated with oxidative damage to cellular components. Antioxidants are therefore frequently examined in vitro for their ability to neutralize free radicals and limit oxidative reactions under controlled experimental conditions [35].

In recent years, researchers have increasingly focused on natural products exhibiting more than one in vitro biochemical activity, such as antioxidant capacity combined with enzyme inhibition. Previous studies have reported that phenolic-rich *Thymus* extracts may display both antioxidant activity and acetylcholinesterase (AChE) inhibition, primarily based on in vitro assays [37,38,39,40], with some observations also reported from in vivo studies [41,42]. Such observations are useful for initial screening purposes but do not allow conclusions regarding biological efficacy or therapeutic relevance without further validation.

The results of the present study suggest that species-specific combinations of polar and apolar metabolites contribute to the observed in vitro antioxidant capacity and AchE-inhibitory activity of *Thymus* extracts. These findings are limited to chemical and enzyme-based assays and should be interpreted as preliminary, serving as a basis for further investigation using more biologically relevant experimental models.

Although the current study did not investigate the influence of edaphic or geological variables on phytochemical diversity, it is worth noting that environmental factors such as soil type, hydrology, and trace element availability are known to shape the chemical composition of wild-growing medicinal plants. In this context, the higher accumulation of phenolic compounds and triterpenic acids observed in species growing in xerothermic or serpentine habitats (e.g., *T. zygioides*, *T. leucotrichus* and *T. jalasianus*) may reflect adaptive metabolic responses to environmental stress conditions such as increased solar radiation, drought, or nutrient imbalance. Such ecological pressures are frequently associated with enhanced production of protective secondary metabolites, including phenolic antioxidants and terpenoids. In this context, previous hydrogeological studies in northern and western Bulgaria have demonstrated the impact of substrate characteristics and groundwater quality on the biogeochemical dynamics of the landscape [43,44]. Furthermore, soil water regimes and evapotranspiration rates, key factors for plant stress responses, have been modeled in loess terrains of northeastern Bulgaria [45]. These abiotic parameters may partially influence the accumulation of phenolic compounds, triterpenes, and essential oil constituents in *Thymus* species, particularly those growing under xerothermic or serpentine conditions. Further integrated ecological and geochemical studies would be valuable to explore such relationships in detail.

## 4. Materials and Methods

### 4.1. Plant Material

Aerial parts of eight *Thymus* species were collected during their flowering period (May–August, 2023) from diverse natural habitats across Bulgaria, with particular attention to endemic and sub-endemic taxa within section *Hyphodromi*. The sampling sites represented a wide ecological gradient—from alpine zones in the Pirin Mountains (up to 2300 m a.s.l.) to lowland serpentine terrains in the Eastern Rhodopes and coastal limestone cliffs near Balchik (Table 6). The plant material was taxonomically verified based on the Flora of Bulgaria and regional identification keys, and voucher specimens were deposited in the herbarium of the Institute of Biodiversity and Ecosystem Research, Bulgarian Academy of Sciences (SOM).

Geographical coordinates and altitudes of the sampling locations were recorded using GPS. Representative species included *T. perinicus* and *T. atticus*, collected from high-mountain localities (Pirin and Slavyanka), *T. comptus* and *T. striatus* from mid-altitude inland regions (Vlahina and Sredna Gora), and *T. zygioides*, *T. leucotrichus*, and *T. jalasianus* from xerothermic and serpentine zones of the Black Sea coast and Eastern Rhodopes. All samples were collected with minimal disturbance to wild populations and air-dried immediately in shaded, ventilated conditions for subsequent analyses.

For each species, the collected plant material was pooled and homogenized prior to extraction, and all extractions and subsequent analyses were performed in triplicate as technical replicates derived from the same pooled biological sample.

### 4.2. Extraction Procedure

Triplicate measurements represent technical replicates and are reported as mean ± standard deviation.

#### 4.2.1. Methanolic (MeOH) Extracts

Approximately 200 mg of dried biomass was placed in 50 mL glass test tubes with teflon-lined phenolic screw caps (Corning Inc., New York, NY, USA) and extracted in triplicate with 20 mL 70% MeOH (VWR International, Radnor, PE, USA) in an ultrasonic bath (Raypa, Barcelona, Spain) for 30 min each. The combined MeOH fractions were evaporated to dryness under vacuum at 50 °C (Heidolph Instruments GmbH & Co. KG, Schwabach, Germany) and the dry extract was dissolved in 1 mL HPLC-grade methanol (Honeywell International Inc., Charlotte, NC, USA) and used for analyses.

#### 4.2.2. Diethyl Ether (Et_2_O) Extracts

Dried plant material (100 mg) was placed in Eppendorf tubes and extracted with 1 mL Et_2_O in an ultrasonic bath for 15 min. At the onset of extraction, 50 μL of nonadecanoic acid (stock solution 1 mg/mL) was added as an internal standard. Following extraction, the Et_2_O fraction was carefully decanted into clean vials and evaporated to dryness under a stream of nitrogen.

### 4.3. Phytochemical Analysis

#### 4.3.1. Derivatization

Prior to GC–MS analysis, Et_2_O extracts were derivatized to improve volatility and stability of the analytes. To achieve this, 100 μL pyridine and 100 μL *N*,*O*-bis-(trimethylsilyl)trifluoroacetamide (BSTFA) were added to each dry extract. The samples were then heated at 60 °C for 2 h to complete the derivatization reaction.

#### 4.3.2. GC–MS Analysis

Gas chromatography–mass spectrometry (GC–MS) was performed using a Thermo Scientific Focus GC system coupled to a Thermo Scientific DSQ mass detector (Thermo Fisher Scientific, Waltham, MA, USA) operating in electron ionization (EI) mode at 70 eV. Separation was achieved using an ADB-5MS capillary column (30 m × 0.25 mm × 0.25 μm).

The chromatographic conditions used for the diethyl ether (Et_2_O) extracts were as follows: 100–180 °C at 15 °C min^−1^, 180–300 °C at 5 °C min^−1^ and 10 min hold at 300 °C. The injector temperature was 250 °C. The flow rate of the carrier gas (Helium) was 0.8 mL min^−1^. The split ratio was 1:10, and 1 μL of the solution was injected.

Analytical conditions followed those described by Berkov et al. [46]. Compound quantification in the Et_2_O fractions was performed in a semi-quantitative manner based on peak area integration and expressed as nonadecanoic acid equivalents, using the internal standard for normalization.

#### 4.3.3. HPLC Analysis

The quantification of individual phenolic acids and flavonoids in extract was achieved by using a Waters HPLC system equipped with UV-VIS detector (Waters, Milford, MA, USA). Each sample was filtered by 0.45 µm PTFE syringe filter (Corning, New York, NY, USA) and 20 µL was injected into a C18 column (Supelco Discovery HS; 5 μm, 25 cm × 4.6 mm) (Merck KGaA, Darmstadt, Germany). Gradient elution (flow rate of 1 mL min^−1^) with 1% acetic acid in water (Solvent A) and methanol (Solvent B) with the following change for Solvent A was applied: 0 to 36 min, Solvent A decreased from 90% to 78%; 36 to 37 min, it decreased from 78% to 70%; 37 to 47 min, it decreased from 70% to 60%; 47 to 58 min, it decreased from 60% to 54%; 58 to 59 min, it decreased from 54% to 40%; 59 to 71 min, it decreased from 40% to 20%; 71 to 72 min, it increased from 20% to 90%; and 72 to 75 min, it was held at 90%. Individual compounds were identified by their retention time, and quantification was carried out by using calibration curves of the following standards: gallic, protocatechuic, vanillic, syringic, *p*-coumaric, and salicylic acids, (+)-catechin, (+)-epicatechin, and hesperidin (detected at λ = 280 nm) and rosmarinic, chlorogenic, caffeic, and ferulic acids, rutin, quercetin, and kaempferol (detected at λ = 360 nm). Identification of phenolic compounds was performed by comparing retention times and UV spectra with those of authentic reference standards analyzed under identical chromatographic conditions. Compounds being listed as ‘NF’ indicates that these analytes were either absent or present at concentrations below the detection limits of the targeted HPLC–UV method used.

#### 4.3.4. Total Phenolic Content

The total phenolic content of the MeOH extracts was determined spectrophotometrically using Folin–Ciocalteu reagent, with gallic acid as the standard. Samples were dissolved to a concentration of 2 mg/mL, and 0.20 mL were mixed with 2 mL of Folin–Ciocalteu reagent (beforehand diluted 10-fold using distilled water) and 1.8 mL of 7.5% Na_2_CO_3_. After 1 h at room temperature, the absorbance of the samples was measured at 765 nm using a spectrophotometer (Jenway 6320D, Cole-Parmer, Stone, Staffordshire, UK) and compared to a blank sample (0.20 mL methanol without extract, 2 mL of Folin–Ciocalteu reagent and 1.8 mL of 7.5% Na_2_CO_3_). All samples were analyzed in triplicate. Results were expressed as gallic acid equivalents (GAE) per gram of dry extract. The total polyphenol content was calculated using the following formula:

$$C=\frac{c\times V}{m}$$

where C is the total content of phenolic compounds (mg g^−1^ plant extract) in GAE; c is the concentration of gallic acid established using the calibration curve in mg mL^−1^; V is the volume of the sample in mL; and m is the weight of the pure plant sample in grams.

### 4.4. Biological Activity

#### 4.4.1. Antiradical Activity

The radical scavenging activity of the MeOH extracts was evaluated using the DPPH assay. Sample solutions were prepared at 10, 20, 50, 100, and 200 µg/mL. Then, 2.5 mL of each sample solution was mixed with 1.0 mL of 0.3 mM DPPH in methanol and incubated in the dark at room temperature for 30 min. The absorbance was measured at 517 nm using a Jenway 6320D visible spectrophotometer (Cole-Parmer, UK) and converted into the percentage antioxidant activity using the following equation:

$$DPPH\;free\;radical\;scavenging\;capacity\;(\%)=\frac{1-\;(Ab\;of\;sample-Ab\;of\;blank)}{Ab\;of\;control}\times 100$$

MeOH (1.0 mL) plus sample solution (2.5 mL) was used as a blank, whereas DPPH solution (1 mL) plus MeOH (2.5 mL) was used as a control. The IC_50_ values (the sample concentration leading to 50% scavenged radicals) were calculated in Prism 3.0 (GraphPad Inc., San Diego, CA, USA) using a sigmoid non-linear regression model. All measurements were performed in triplicate, and results are reported as means ± standard deviation (SD). Butylated hydroxytoluene (BHT) was used as a positive control in the DPPH assay, as commonly applied in comparable antioxidant studies, while Trolox was not included in the present experimental design.

#### 4.4.2. Acetylcholinesterase-Inhibitory Activity

A microplate-based assay was employed to determine the AchE-inhibitory activity of the extracts, following a modified version of the method originally described by Ellman et al. [47] and López et al. [48]. First, all samples were dissolved in a phosphate buffer (PBS) (8 mM K_2_HPO_4_, 2.3 mM NaH_2_PO_4_, 0.15 M NaCl, pH 7.5), aided by the addition of MeOH, but no more than 5% in the final solutions. Next, they were diluted using PBS to provide the concentration range needed. Seven different concentrations from 0.01 to 1 mg/mL were tested. Acetylthiocholine iodide (ATCI) in a solution with 5,5′-dithiobis(2-nitrobenzoic acid) (DTNB) was used as a substrate for AChE from *Electrophorus electricus* (Sigma-Aldrich, Taufkirchen, Germany) (0.04 M Na_2_HPO_4_, 0.2 mM DTNB, 0.24 mM ATCI, pH 7.5).

Then, 50 µL of AChE (0.25 U/mL) dissolved in PBS and 50 µL of the tested sample solution was added to the wells. The incubation of the plates was performed at room temperature for 30 min. Then, 100 µL of the substrate solution was added to start the enzymatic reaction. The absorbances were read using a microplate reader (BIOBASE, ELISA-EL10A, Jinan, China) at 405 nm after 5 min. Enzyme activity was calculated as an inhibition percentage compared to an assay including a buffer instead of an inhibitor. Galanthamine was used as a positive control.

All extracts were initially screened for acetylcholinesterase-inhibitory activity at concentrations ranging from 0.01 to 1 mg/mL. For samples showing detectable inhibition within this range, additional concentrations up to 3 mg/mL were included to allow determination of IC_50_ values.

All data were analyzed with the software package Prism version 3.0 (Graph Pad Inc., San Diego, CA, USA). The IC_50_ values (half maximal inhibitory concentration) were determined from three independent measurements (*n* = 3), and the results are presented as means ± SD.

## 5. Conclusions

The present study provides a comprehensive phytochemical characterization and evaluation of the in vitro biological activity of eight *Thymus* species from Bulgaria, with a particular focus on taxa belonging to section *Hyphodromi*. GC–MS and HPLC analyses revealed species-specific metabolite profiles dominated by phenolic acids, flavonoids, triterpenic acids, and monoterpenes.

The methanolic extracts exhibited pronounced antioxidant capacity in a chemical radical scavenging assay, which correlated with their total phenolic content. Moderate acetylcholinesterase-inhibitory activity was observed only for *T. zygioides* and *T. leucotrichus* under in vitro conditions.

These findings should be interpreted strictly within the limitations of enzyme-based and chemical assays and do not imply neuroprotective, neuroactive, or therapeutic effects. Nevertheless, the presented data contribute valuable information on the chemodiversity and biochemical properties of endemic and sub-endemic Bulgarian *Thymus* species and provide a foundation for future studies employing cellular, in vivo, and mechanistic approaches to assessing biological relevance.

## Figures and Tables

**Table 1:** Compounds identified in the diethyl ether extracts of the studied *Thymus* species by GC–MS.

Compounds	RI	*T.* *atticus*	*T.* *perinicus*	*T.* *striatus*	*T.* *comptus*	*T. aznavourii*	*T. zygioides*	*T.* *leucotrichus*	*T. jalasianus*
**exo-Borneol**	1220						9.11 ± 2	9.82 ± 0.7	
**Thymoquinone**	1250						3.77 ± 0.6	6.62 ± 0.5	
**Glycerol**	1289	3.56 ± 0.7	1.89 ± 0.1	3.82 ± 0.9	3.12 ± 1.4	2.40 ± 0.9	2.51 ± 1.4	1.74 ± 0.3	2.26 ± 0.4
**Thymol**	1322	15.56 ± 2	41.27 ± 5.2	23.23 ± 2.8	7.62 ± 2	11.78 ± 7.2	41.25 ± 9.5	250.59 ± 1.3	4.92 ± 1.8
**Carvacrol**	1339	4.89 ± 0.4	22.24 ± 1.1	7.48 ± 2.3	7.55 ± 6.6	8.69 ± 4.4	147.58 ± 15	31.13 ± 3	2.00 ± 0.8
***tert*** **-Butylhydroquinone**	1552	13.71 ± 7.3	10.77 ± 0.9		8.66 ± 7.1	11.09 ± 0.4	5.12 ± 2.1	6.11 ± 1.1	6.86 ± 1.5
**1-Dodecanol**	1558	16.77 ± 9.6		13.36 ± 4.5		11.29 ± 2.3	9.14 ± 2.2	6.77 ± 0.6	8.65 ± 1.3
**Hexadecanoic acid (C16:0)**	2040	34.35 ± 2.2	28.97 ± 2	45.21 ± 12	38.68 ± 3.9	24.36 ± 2.9	30.73 ± 1.3	39.37 ± 0.8	28.52 ± 3.5
**Octadecadienoic acid (C18:2)**	2089	8.26 ± 5.5	21.05 ± 1.8	10.53 ± 2.0	15.81 ± 6.2	6.16 ± 1.1	7.96 ± 2.1	11.06 ± 1.6	10.48 ± 2.2
**Octadecenoic acid (C18:1)**	2222	11.26 ± 2.7	52.63 ± 4.4	26.36 ± 1.8	39.83 ± 16	15.41 ± 2.7	28.31 ± 2.5	27.64 ± 4.5	26.21 ± 2.4
**Octadecanoic acid (C18:0)**	2246	9.80 ± 4.4	6.47 ± 1.3	8.21 ± 0.9	8.11 ± 2.2	7.98 ± 2.1	8.84 ± 1.7	8.30 ± 1.3	8.21 ± 1.1
**Glycerol monopalmitate**	2607	10.62 ± 3.3	6.46 ± 0.3	7.17 ± 0.8	9.52 ± 2.7	8.15 ± 2.6	10.20 ± 2.2	3.99 ± 0.2	13.88 ± 2.8
**Glycerol monostearate**	2806	7.17 ± 1.6				1.22 ± 0.5		0.93 ± 0.2	6.67 ± 1.5
**Naringenin**	2890				16.36 ± 2.5		6.58 ± 0.3	4.66 ± 0.9	
**β-Sitosterol**	3335	4.45 ± 1			5.59 ± 1.9	3.00 ± 1.5	10.13 ± 3.0	4.16 ± 1.9	9.30 ± 3.1
**Methoxyflavone**	3359					3.3 ± 0.4			13.83 ± 4.5
**Uvaol**	3434						2.54 ± 0.7		5.22 ± 1.8
**Oleanolic acid**	3523	74.87 ± 12	32.75 ± 3.3	112.14 ± 5.7	85.72 ± 15	76.52 ± 11	88.54 ± 3.6	21.78 ± 2.2	99.62 ± 7.3
**Ursolic acid**	3530	63.16 ± 14	77.96 ± 2.3	76.52 ± 7.4	125.37 ± 21	79.30 ± 12	142.29 ± 22	49.44 ± 4.8	207.23 ± 13
**Micromeric acid**	3642		11.86 ± 2.4				7.30 ± 1.7	6.95 ± 1.5	14.62 ± 1.1

Legend: RI—retention index.

**Table 2:** Phenolic compounds identified in the methanolic extracts of the studied *Thymus* species by HPLC.

Compounds	Content mg/g Dry Weight in the Studied Plant Species							
	*T. atticus*	*T. perinicus*	*T. striatus*	*T. comptus*	*T. aznavourii*	*T. zygioides*	*T. leucotrichus*	*T. jalasianus*
**Gallic acid**								
**Protocatechuic acid**								
**(+)-Catechin**								
**Chlorogenic acid**								
**Vanillic acid**								
**Caffeic acid**								
**Syringic acid**								
**(−)-Epicatechin**								
***p*** **-Coumaric acid**	0.46							
**Ferulic acid**	**4.96**			0.69				
**Salicylic acid**	6.68	3.54	1.88		8.12	1.87	1.71	3.50
**Rutin**	**2.03**	1.16	0.49	1.55	2.58	0.88	3.32	1.37
**Hesperidin**	5.88							1.27
**Rosmarinic acid**	**4.65**	1.51	1.85	1.93	2.20	1.72	1.64	2.60
**Quercetin**	0.19	0.07	0.12	0.05	**0.95**	0.38	0.16	0.37
**Kaempferol**								

Legend: Compounds whose contents are not reported in mg/g dry weight were not detected or were present below the detection limits of the targeted HPLC–UV method; major constituents are highlighted in bold.

**Table 3:** Total phenolic contents and radical scavenging potential of the studied *Thymus* species.

*Thymus* Species	Total Phenolic Content [mg GAE/g Dry Extract]	DPPH Radical Scavenging Activity [IC_50_, μg/mL]
*T. atticus*	14.88 ± 0.38	23.67 ± 3.17
*T. perinicus*	5.07 ± 0.30	43.15 ± 2.32
*T. striatus*	14.82 ± 1.70	28.09 ± 1.6
*T. comptus*	14.55 ± 0.30	24.75 ± 3.45
*T. aznavourii*	15.49 ± 0.83	25.60 ± 2.32
*T. zygioides*	5.90 ± 1.23	45.61 ± 1.6
*T. leucotrichus*	15.97 ± 1.37	29.80 ± 2.6
*T. jalasianus*	15.80 ± 0.05	24.41 ± 0.36
Rosmarinic acid (pc)		5.04 ± 0.3
Butylated hydroxytoluene (pc)		12.62 ± 0.56

GAE—gallic acid equivalents; pc—positive control. BHT was used as a positive control.

**Table 4:** Acetylcholinesterase inhibition percentages and IC_50_ values for the tested *Thymus* MeOH extracts.

*Thymus* Species	Inhibition Percentage of 1 mg/mL	Inhibition Percentage of 3 mg/mL	AChE Activity [IC_50_, mg/mL]
*T. atticus*	1.55 ± 0.88	nt	>1
*T. perinicus*	0	nt	>1
*T. striatus*	0	nt	>1
*T. comptus*	2.66 ± 1.78	nt	>1
*T. aznavourii*	0	nt	>1
*T. zygioides*	16.02 ± 1.27	58.98 ± 4.55	2.40 ± 0.08
*T. leucotrichus*	16.41 ± 0.68	53.02 ± 4.85	3.07 ± 0.22
*T. jalasianus*	0	nt	>1
galanthamine (pc)			1.41 ± 0.08 μM (0.40 ± 0.02 μg/mL)

pc—positive control; 0—no inhibition percentage detected; nt—not tested.

**Table 5:** Metabolites identified in the methanolic extracts of *T. zygioides* and *T. leucotrichus*.

Compounds	RT	Methanolic Extracts [Area %]	
		*T. zygioides*	*T. leucotrichus*
**Glycerol**	1289	2.6 ± 0.7	2.78 ± 1.5
**Phosphoric acid**	1293	0.5 ± 0.1	0.64 ± 0.1
**Thymol**	1322	**2.2 ± 0.8**	**7.64 ± 0.1**
**Carvacrol**	1339	**21.7 ± 8.8**	**4.42 ± 4.2**
**Malic acid**	1469	1.3 ± 0.4	1.17 ± 0.4
**meso-Erythritol**	1501	1.8 ± 0.5	1.01 ± 0.5
***tert*** **-Butylhydroquinone**	1596	0.8 ± 0.3	0.54 ± 0.2
**4-Hydroxybenzoic acid**	1635	0.1 ± 0	0.1 ± 0
**Arabitol**	1746	0.9 ± 0.3	2.22 ± 0.9
**Fructose 1**	1789	0.7 ± 0.2	1.1 ± 0.3
**Fructose 2**	1800	0.7 ± 0.3	1.97 ± 0.7
**Fructose 3**	1803	0.4 ± 0.1	2.33 ± 0.6
**Protocatechuic acid**	1813	0.1 ± 0	0.4 ± 0.1
**Quinic acid**	1843	0.8 ± 0.3	2.13 ± 0.7
**Glucose**	1882	2.71 ± 0.4	3.55 ± 0.3
**Hexadecanoic acid**	2040	2.1 ± 0.8	4.22 ± 0.5
***myo*** **Inositol**	2090	1.6 ± 0.1	1.77 ± 0.9
**Caffeic acid**	2141	2.0 ± 0.7	0.51 ± 0.4
**Ferulic acid**	2055	0.1 ± 0	0.1 ± 0
**Octadecadienoic acid**	2089	0.3 ± 0.1	1.07 ± 0.1
**Octadecatrienoic acid**	2218	2.1 ± 0.1	0.36 ± 0.1
**Sucrose**	2628	1.6 ± 0.4	3.33 ± 0.4
**1-Monopalmitin**	2635		2.18 ± 1
**Glycerol monostearate**	2806		1.1 ± 0.8
**Chlorogenic acid**	3100	0.4 ± 0.1	0.13 ± 0.1
**4-O-Feruloylquinic acid**	3138	0.65 ± 0.2	0.20 ± 0.1
**3-O-Feruloylquinic acid**	3162	0.28 ± 0.1	0.18 ± 0.1
**β-Sitosterol**	3335	1.1 ± 0.1	1.27 ± 0.2
**Rosmarinic acid**	3510	2.9 ± 0.3	2.2 ± 0.5
**Oleanolic acid**	3525	**4.54 ± 1.1**	**9.25 ± 0.2**
**Ursolic acid**	3530	**9.72 ± 0.45**	**15.8 ± 0.7**
**Micromeric acid**	3642		1.54 ± 0.2

Legend: major bioactive constituents are highlighted in bold.

**Table 6:** Studied *Thymus* species and geographical conditions of sampling sites.

Sample	Species	Collection Site	Altitude (m)	Coordinates (°N, °E)
T1	*Thymus atticus* Čelak.	Slavyanka Mts., near Paril village	760	41.4294, 23.7250
T2	*Thymus perinicus* (Velen.) Jalas	Pirin Mts., near Vlahini Lakes	2300	41.7560, 23.2800
T3	*Thymus striatus* Vahl	Sredna Gora Mts., near Banya village (Karlovo region)	900	42.5778, 24.7484
T4	*Thymus comptus* Friv.	Vlahina Mts., between Selishte and Logodazh	900	41.8536, 22.9230
T5	*Thymus aznavourii* Velen.	Eastern Rhodope Mts., near Sladun village	120	41.8344, 26.4820
T6	*Thymus zygioides* Griseb.	Coastal area near Balchik	300	43.3000, 28.1000
T7	*Thymus leucotrichus* Halácsy	Rhodope Mts.	800	41.5000, 24.3330
T8	*Thymus jalasianus* Stoyanov & Marinov	Eastern Rhodope serpentine area (between Fotinovo and Sladun)	650	41.8350, 25.4100

## Data Availability

The data presented in this study are available in this article and in the Supplementary Materials. Additional data supporting the findings of this study are available from the corresponding author upon reasonable request.

## Supplementary Materials

The following supporting information can be downloaded at: https://www.mdpi.com/article/10.3390/plants15060927/s1. Figure S1. HPLC fingerprints of standards; Figure S2. HPLC fingerprints of *Thymus aznavourii*; Figure S3. HPLC fingerprints of *Thymus atticus*; Figure S4. HPLC fingerprints of *Thymus jalasianus*; *Thymus perinicus*; Figure S5. HPLC fingerprints of *Thymus perinicus*; Figure S6. HPLC fingerprints of *Thymus comptus*; Figure S7. HPLC fingerprints of *Thymus zygioides*; Figure S8. HPLC fingerprints of *Thymus leucotrichus*; Figure S9. HPLC fingerprints of *Thymus striatus*.
