# Supplementary material for: Species-Specific Metabolite Profiles and Biological Activities of Bulgarian Thymus Species from Section Hyphodromi
Source: Plants (Basel). 2026 Mar 17;15(6):927. doi: 10.3390/plants15060927 (PMC13030227; doi:10.3390/plants15060927)
Supplement: Supplementary file 1 [file plants-15-00927-s001.zip › plants-4150919-supplementary.pdf]

Supplementary

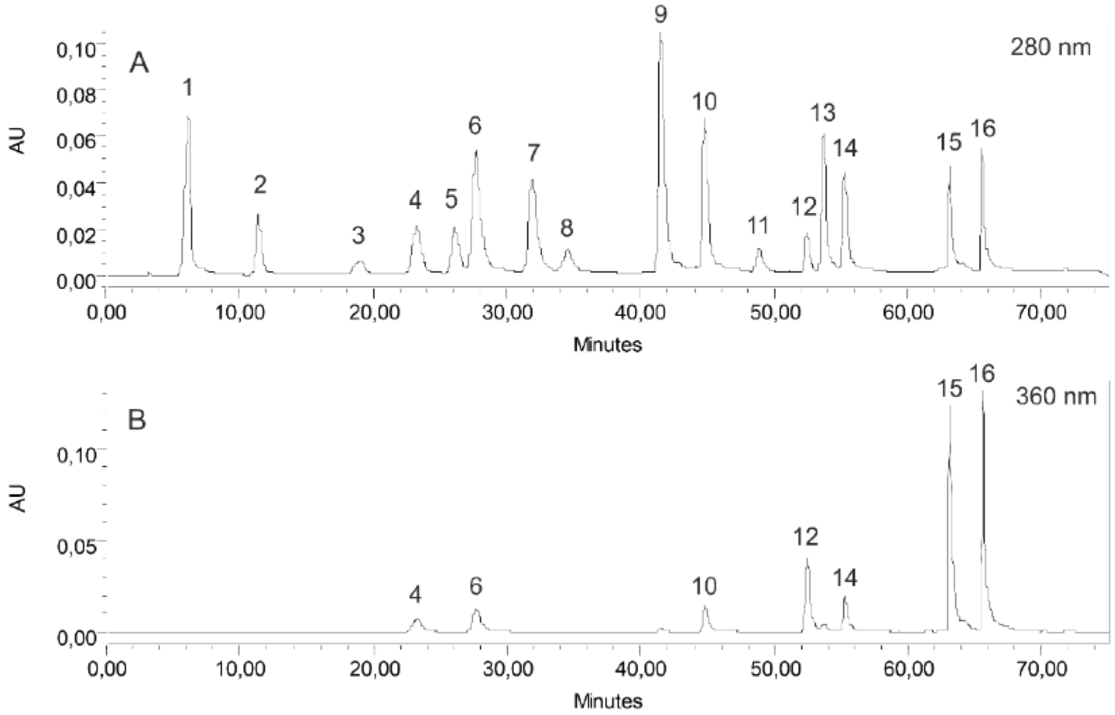

Figure S1. HPLC fingerprints of standards at 280 nm (A) and 360 nm (B): 1 - Gallic acid; 2 - Protocatehuic acid; 3 - (+)-Catechin; 4 - Chlorogenic acid; 5 - Vanillic acid; 6 - Caffeic acid; 7 - Syringic acid; 8 - (-)-Epicatechin; 9 - p-Coumaric acid; 10 - Ferulic acid; 11 - Salicylic acid; 12 – Rutin; 13 – Hesperidin; 14 - Rosmarinic acid; 15 – Quercetin; 16 - Kaempherol.

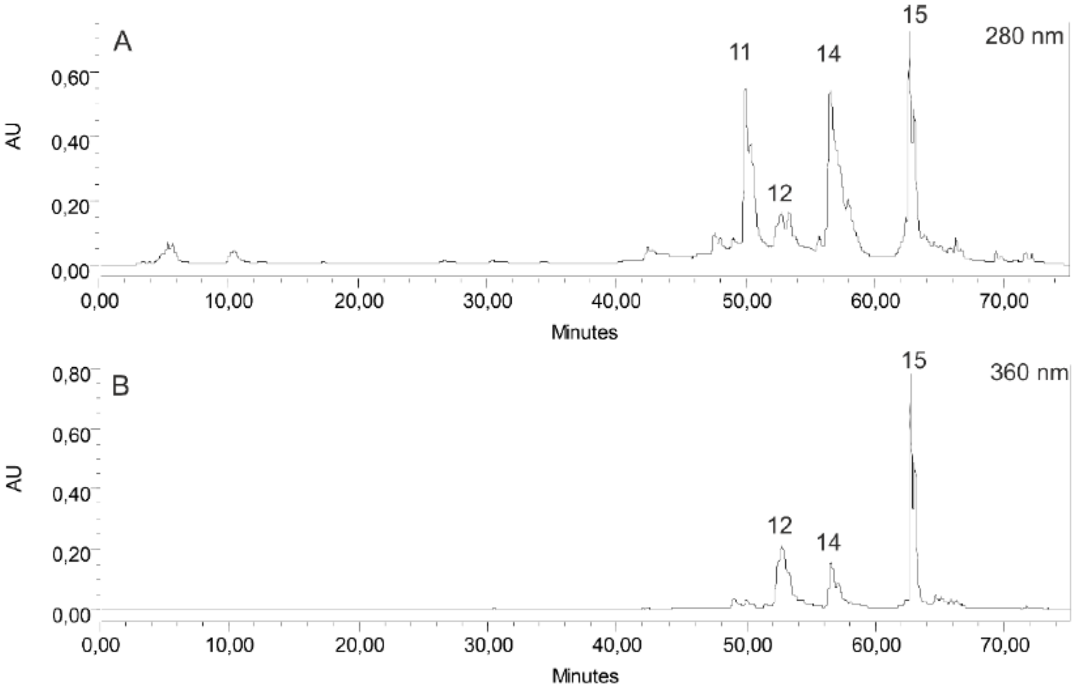

Figure S2. HPLC fingerprints of *Thymus aznavourii* at 280 nm (A) and 360 nm (B): 11 - Salicylic acid; 12 – Rutin; 14 - Rosmarinic acid; 15 – Quercetin.

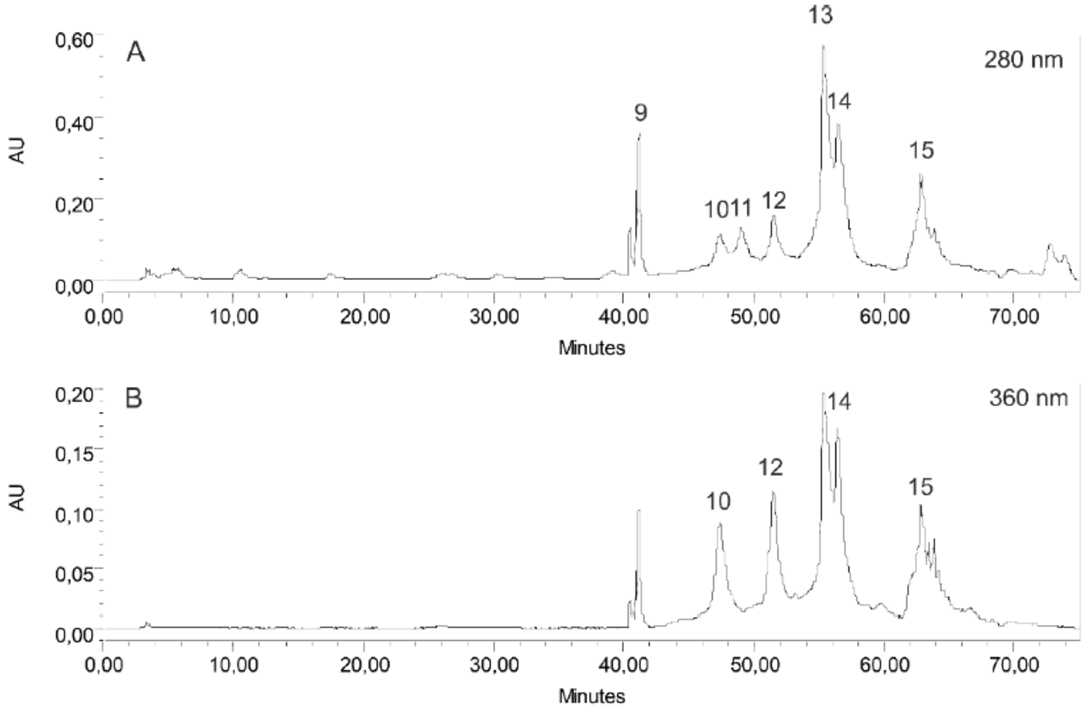

Figure S3. HPLC fingerprints of *Thymus atticus* at 280 nm (A) and 360 nm (B): 9 - p-Coumaric acid; 10 - Ferulic acid; 11 - Salicylic acid; 12 – Rutin; 13 – Hesperidin; 14 - Rosmarinic acid; 15 – Quercetin.

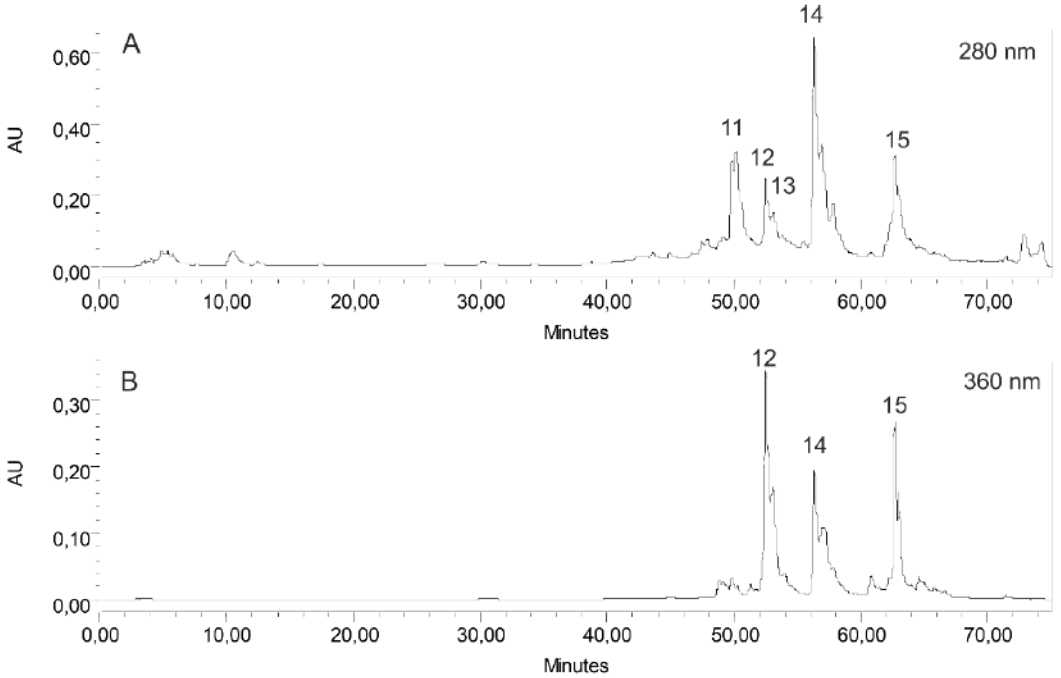

Figure S4. HPLC fingerprints of *Thymus jalasianus* at 280 nm (A) and 360 nm (B): 11 - Salicylic acid; 12 – Rutin; 13 – Hesperidin; 14 - Rosmarinic acid; 15 – Quercetin.

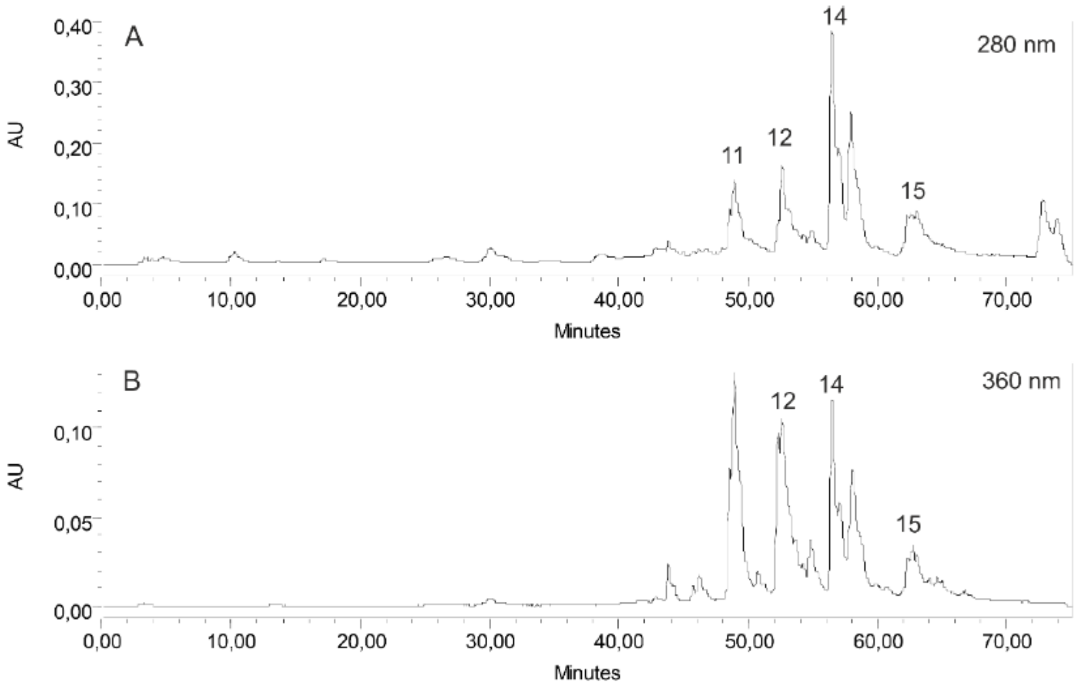

Figure S5. HPLC fingerprints of *Thymus perinicus* at 280 nm (A) and 360 nm (B): 11 - Salicylic acid; 12 – Rutin; 14 - Rosmarinic acid; 15 – Quercetin.

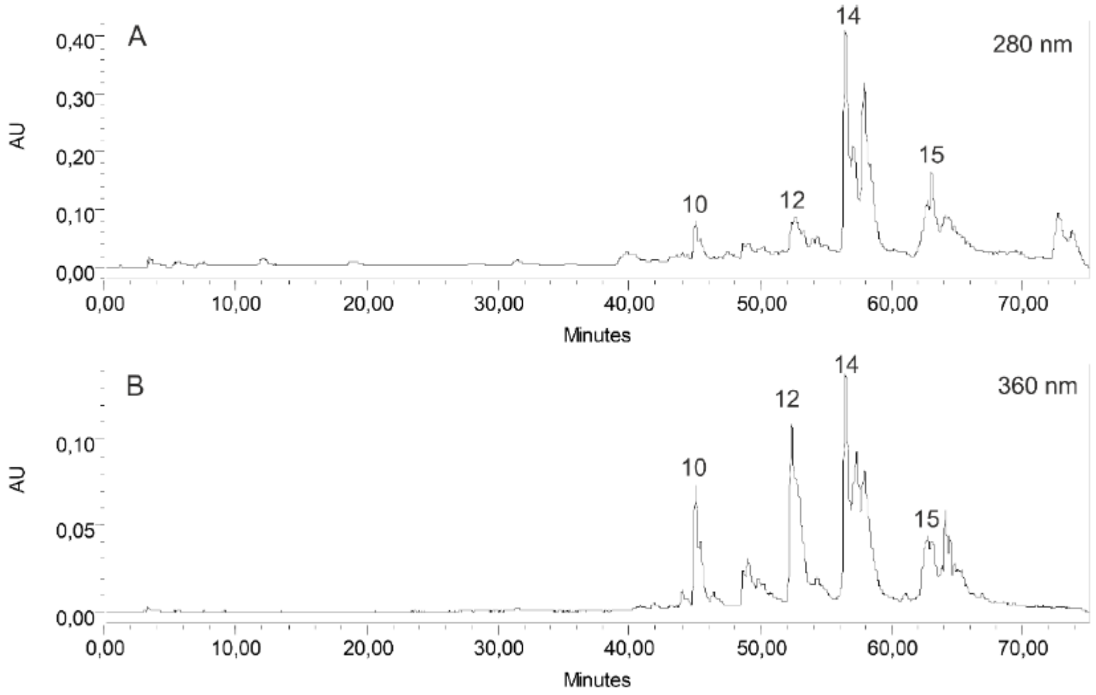

Figure S6. HPLC fingerprints of *Thymus comptus* at 280 nm (A) and 360 nm (B): 10 - Ferulic acid; 12 – Rutin; 14 - Rosmarinic acid; 15 – Quercetin.

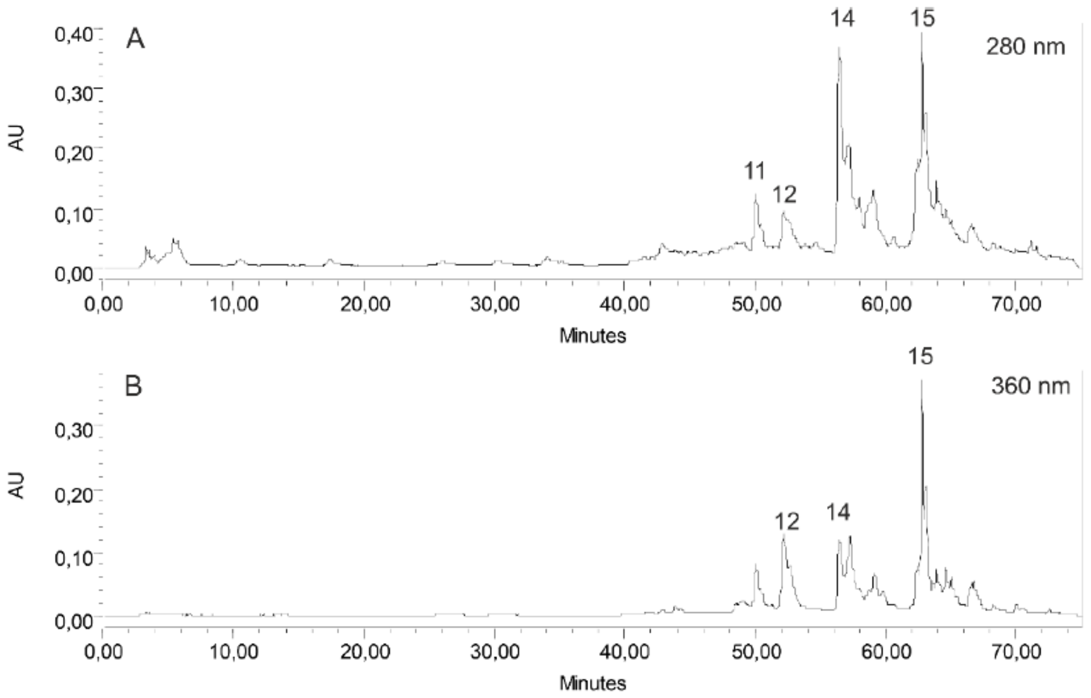

Figure S7. HPLC fingerprints of *Thymus zygioides* at 280 nm (A) and 360 nm (B): 11 - Salicylic acid; 12 – Rutin; 14 - Rosmarinic acid; 15 – Quercetin.

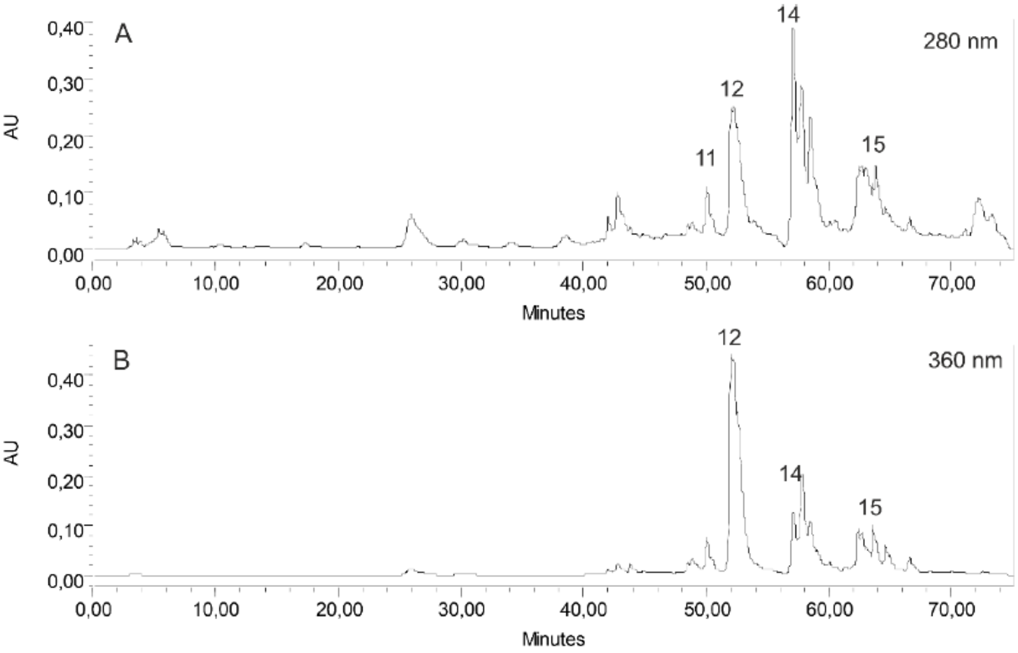

Figure S8. HPLC fingerprints of *Thymus leucotrichus* at 280 nm (A) and 360 nm (B): 11 - Salicylic acid; 12 – Rutin; 14 - Rosmarinic acid; 15 – Quercetin.

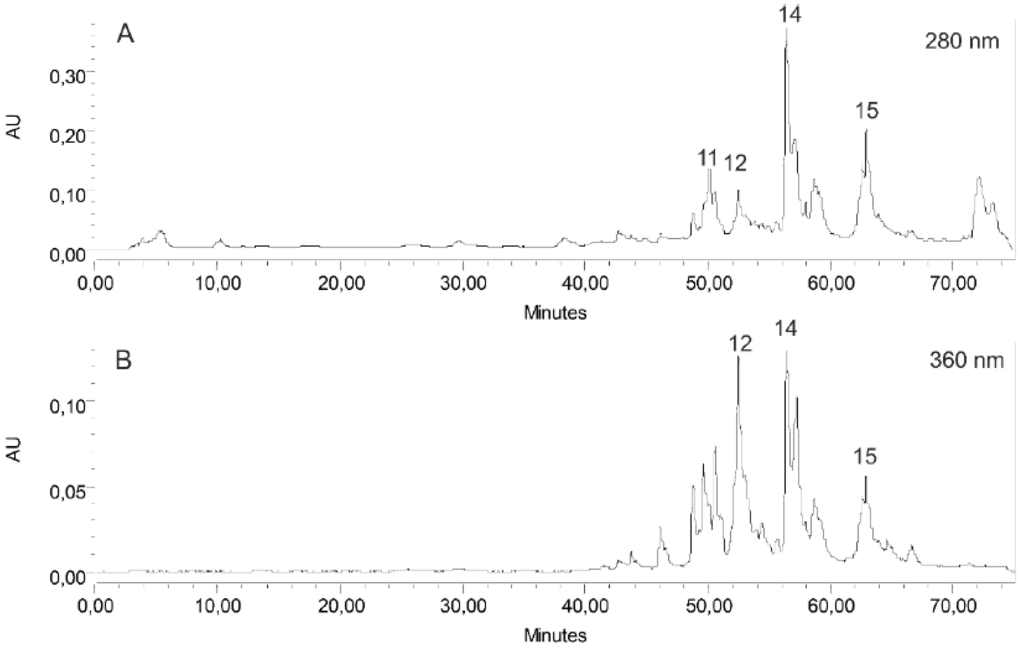

Figure S9. HPLC fingerprints of *Thymus striatus* at 280 nm (A) and 360 nm (B): 11 - Salicylic acid; 12 – Rutin; 14 - Rosmarinic acid; 15 – Quercetin.
